# 开放性实验:聚乙烯亚胺修饰碳点的合成、纯化与表征

**DOI:** 10.3724/SP.J.1123.2023.12017

**Published:** 2024-04-08

**Authors:** Rui LI, Guanhong XU, Hailin YU, Yao CEN, Yan PENG, Fanli SHEN, Fangdi WEI

**Affiliations:** 1.南京医科大学, 江苏 南京 211166; 1. Nanjing Medical University, Nanjing 211166, China; 2.江苏省食品药品监督检验研究院, 江苏 南京 210019; 2. Jiangsu Institute for Food and Drug Control, Nanjing 210019, China

**Keywords:** 开放性实验, 聚乙烯亚胺, 碳点, 荧光, 纯化方法, open experiment, polyethyleneimine (PEI), carbon dots (CDs), fluorescence, purification method

## Abstract

开放性实验是培养拔尖创新人才的有效手段。我们基于学生兴趣、科研热点和实验室条件,设计了实验方案。实验采用一步水热法,以柠檬酸(CA)为碳源,聚乙烯亚胺(PEI)为修饰剂,合成PEI修饰碳点(PEI-CDs)。首先,将CA、PEI在0.1 mol/L的盐酸中超声至完全溶解,混匀后转移到高压反应釜中,放入烘箱130 ℃反应2 h,制得PEI-CDs原液;然后将冷却至室温的原液旋转蒸发浓缩至2 mL,并用无水乙醇沉淀、洗涤;最后,将所得沉淀放入真空干燥箱,70 ℃干燥12 h得到PEI-CDs粉末。利用紫外-可见分光光度计、荧光分光光度计、红外光谱仪和透射电镜对所合成的PEI-CDs进行表征。结果表明,所采用的无水乙醇沉淀纯化方法简单、快速、经济、绿色,制得的PEI-CDs水溶性好,发光性能优异,大小均一,稳定性高。通过该开放性实验,学生不仅熟悉了大型仪器的使用,而且增强了科学研究的兴趣。

## Abstract (en)

Open experiments are an effective means of cultivating top-notch innovative talents. Based on student interests, research hotspots and our laboratory conditions, an experimental scheme was designed. In this experiment, polyethyleneimine modified carbon dots (PEI-CDs) were prepared via a one-step hydrothermal method using citric acid (CA) as the carbon source and PEI as the surface passivator. First, CA and PEI were completely dissolved in 0.1 mol/L HCl and transferred into an autoclave. The autoclave was heated to 130 ℃ for 2 h. PEI-CDs solution was obtained. After cooling to room temperature, the solution was concentrated to 2 mL by rotary evaporation. Finally, the PEI-CDs were precipitated, washed with ethanol, and dried under vacuum at 70 ℃ for 12 h. The obtained PEI-CDs were characterized by fluorescence spectrophotometry, absorption spectrophotometry, infrared spectrometry, and transmission electron microscopy. The results indicated that anhydrous-ethanol precipitation is a simple, rapid, economical, and green purification method. The as-prepared PEI-CDs had unique properties, such as good water solubility, high luminescence, uniform particle sizes, and good stability. Through this open experiment, students can not only master the operation of large-scale instruments but also enhance their interest in scientific research.

仪器分析是利用各种现代仪器对物质进行检测的方法,是化学研究乃至科学研究的眼睛,是高校多个专业的基础课程^[1]^。仪器分析的多学科交叉性、知识更新快速性、科学前沿紧密性等特点使其在培养拔尖创新人才方面具有得天独厚的优势^[2]^。然而,大型仪器价格贵、成本高,很难开展大规模的实验教学。本项目以开放性实验为载体,将新型纳米材料的合成、纯化及表征与仪器分析实验结合起来,引导学生探索科学研究的前沿,掌握大型仪器的使用,形成创新意识,强化研究能力。

碳点(carbon dots, CDs)是粒径小于10 nm的近球形零维碳质骨架和表面基团构成的荧光纳米微粒。它具有光学性质稳定、生物相容性好、细胞毒性低、易于进行表面修饰等优点,已逐渐成为荧光纳米微粒领域的理想材料,被广泛应用于生物成像、生物传感、化学探针、药物递送、抗菌、色谱分离和光催化等领域^[3⇓⇓⇓⇓-8]^。自从2006年,Sun等^[9]^报道CDs以来,科研人员提出了各种优化CDs性能的方法。其中,氮元素掺杂是调节CDs内在结构性能、优化CDs性质的有效手段,可提高CDs荧光性能及量子产率(photoluminescent quantum yield, PLQY)^[10⇓⇓-13]^。

聚乙烯亚胺(polyethyleneimine, PEI)是一种含有大量-NH_2_的水溶性高分子聚合物,其外端的-NH_2_可与有机化合物的含氧或含氮官能团形成氢键;同时,PEI带有正电荷,可与带有负电荷的分子如DNA、RNA产生静电相互作用形成复合物;另外,PEI还是一种良好的转染试剂^[14,15]^。2012年,Liu等^[16]^制备出了一种PEI修饰CDs(PEI-CDs),随后PEI-CDs的相关研究不断深入^[17⇓⇓⇓-21]^。研究表明,PEI-CDs的纯化方法对其合成产率、光学性能和后续使用具有非常重要的影响。

本实验以柠檬酸(citric acid, CA)为碳源,PEI为修饰剂,采用一步水热法合成PEI-CDs,并探索出一种简单、快速、经济、绿色的纯化方法,提高了PEI-CDs的产率和PLQY。

本实验从选题、设计、实施、总结,坚持“学生为主,教师为辅”的原则,充分发挥学生的主动性,取得了良好的教学效果。

## 1 实验部分

### 1.1 仪器、试剂与材料

AY220电子分析天平(精密度0.0001 g;SHIMADZU,日本); KQ5200型超声波清洗器(昆山市超声仪器有限公司); DZG-6050型真空干燥箱(上海森信实验仪器有限公司); YRE-5299旋转蒸发器(巩义市予华仪器有限责任公司); SHB-Ⅲ循环水式真空泵(南京科尔仪器设备有限公司); TG16-WS台式高速离心机(湖南湘仪实验室仪器开发有限公司); DHG-9240A电热恒温鼓风干燥箱(上海精宏实验设备有限公司); ZF-1型三用紫外线分析仪(上海和勤分析仪器有限公司); F-4600荧光分光光度计(HITACHI,日本); UV-2450紫外-可见分光光度计(SHIMADZU,日本); Tensor红外光谱仪(Bruker,德国); JEM-2100透射电镜(TEM, JEOL,日本); X射线光电子能谱仪(XPS, Thermo Fisher,美国); PURELAB纯水系统(Pall,美国)。

CA(C_6_H_8_O_7_, 99.5%)和无水乙醇(国药集团化学试剂有限公司); PEI(M.W. 1800, 99%,上海麦克林生化有限公司);盐酸(HCl,质量分数36.0%~38.0%,上海凌峰化学试剂有限公司);硫酸奎宁(C_40_H_48_N_4_O_4_·H_2_SO_4_·2H_2_O, 98.6%)和溴化钾(KBr,光谱纯)购自上海阿拉丁生化科技有限公司。

### 1.2 PEI-CDs的合成

本实验采用“一步水热法”合成PEI-CDs。首先,配制0.1 mol/L的HCl,待用;然后,称取0.50 g CA和0.25 g PEI溶于7 mL的盐酸中,超声至完全溶解混匀后转移到30 mL以聚四氟乙烯为内衬的不锈钢高压反应釜中;最后,将高压反应釜密封,放入真空干燥箱中于130 ℃反应2 h。

### 1.3 PEI-CDs的纯化

采用无水乙醇沉淀的纯化方法。将合成的PEI-CDs反应液冷却到室温,在60 ℃下进行旋转蒸发、浓缩至2 mL左右,加入无水乙醇产生沉淀,然后将其在10000 r/min的条件下离心分离10 min,吸除上清液,沉淀用无水乙醇反复洗涤提纯,最后将所得沉淀放入70 ℃烘箱中真空干燥12 h,得到淡黄色粉末状产物。

### 1.4 PEI-CDs的表征

#### 1.4.1 PEI-CDs的光学性能表征

将PEI-CDs配制成1 mg/mL的水溶液,采用荧光分光光度计和紫外-可见分光光度计,在200~650 nm范围内对其激发、发射和吸收性能进行研究。同时,拍摄该溶液在日光和365 nm紫外灯照射下的图片。

PEI-CDs的PLQY采用参比法测定^[22]^,选择硫酸奎宁(*φ*_s_=0.544)作参比物。配制PEI-CDs和硫酸奎宁溶液时,溶剂分别为超纯水和0.1 mol/L的稀硫酸。首先配制1 mg/mL的PEI-CDs和硫酸奎宁贮备液,在紫外-可见分光光度计上测量其在激发波长350 nm处的吸光度*A*;然后依据朗伯-比尔定律,稀释成5份不同浓度的溶液(*A*<0.05);最后测定5份溶液在350 nm下的积分荧光强度*F*。

以*A*为横坐标,*F*为纵坐标,绘制两者的PLQY标准曲线,按式(1)计算PEI-CDs的PLQY:


(1) $$\varphi_{x}=\varphi_{s}\cdot \frac{k_{x}}{k_{s}}\cdot {\left((|\frac{n_{x}}{n_{s}}|)\right)}^{2}$$

式中*φ*_x_和*φ*_s_表示待测物质和参比物质的PLQY; *k*_x_和*k*_s_分别表示待测物质和参比物质的PLQY标准曲线的斜率;*n*_x_和*n*_s_分别表示待测物质和参比物质所用溶剂的折射率。

#### 1.4.2 PEI-CDs的化学组成、尺寸与形貌表征

采用KBr压片法制样,扫描PEI-CDs的傅里叶变换红外光谱图,确认其化学组成。利用XPS,对PEI-CDs的元素组成和化学键类型进行表征。利用TEM,分析PEI-CDs的尺寸和形貌。

#### 1.4.3 PEI-CDs的稳定性表征

将PEI-CDs配制成1 mg/mL的水溶液,置于3 mL离心管中,用于考察PEI-CDs的光照稳定性和放置稳定性。

在光照稳定性实验中,将上述离心管放置于ZF-1型三用紫外线分析仪中,用365 nm紫外光连续照射,每隔一定时间(0、 0.5、 1、 1.5、 2、 3、 4、 5、 6、 7、 8、 10、 12、 14、 16、 18 h)取出其中一份,测定荧光强度。平行测定3次。

在放置稳定性实验中,将上述离心管放置于冰箱(4 ℃)保存,每隔一定时间(0、 1、 2、 3、 4、 5、 6、 7、 8、 10、 12、 24、 36、 48 h)取出其中一份,测定荧光强度。平行测定3次。

## 2 结果与讨论

### 2.1 PEI-CDs纯化方法的优化

目前所报道的PEI-CDs纯化方法主要是透析后进行冷冻干燥^[16⇓⇓-19]^或直接使用^[20,21]^。这些方法的缺点如下:(1)透析耗时长(一般≥24 h),而且期间需不断换水;(2)透析过程中有CDs渗漏,导致CDs损失,CDs的产率很低;(3)若冷冻干燥,则不仅需要相关设备,而且过程非常耗时;(4)若透析后直接使用,则CDs的浓度未知,很难保证后续实验的重复性和重现性。

本实验采用无水乙醇沉淀的方法纯化所合成的PEI-CDs,纯化方法简单、快速、经济、绿色。同时,在本实验条件下,可制得0.3465 g PEI-CDs粉末(CA投料量0.4951 g),产率高达69.80%。

### 2.2 PEI-CDs的表征结果

#### 2.2.1 荧光光谱和吸收光谱表征

图1为PEI-CDs的荧光光谱图和紫外-可见光谱图。由图1a可见,PEI-CDs的激发光谱在340~380 nm范围内连续分布,最大发射波长为450 nm。PEI-CDs水溶液在日光下呈现透明色(图1a内嵌左图),在365 nm紫外灯照射下发射蓝色荧光(图1a内嵌右图)。从图1b中可以看出,PEI-CDs在365 nm处有明显的紫外吸收峰,表明PEI-CDs粒径分布均匀。

**图 1:**
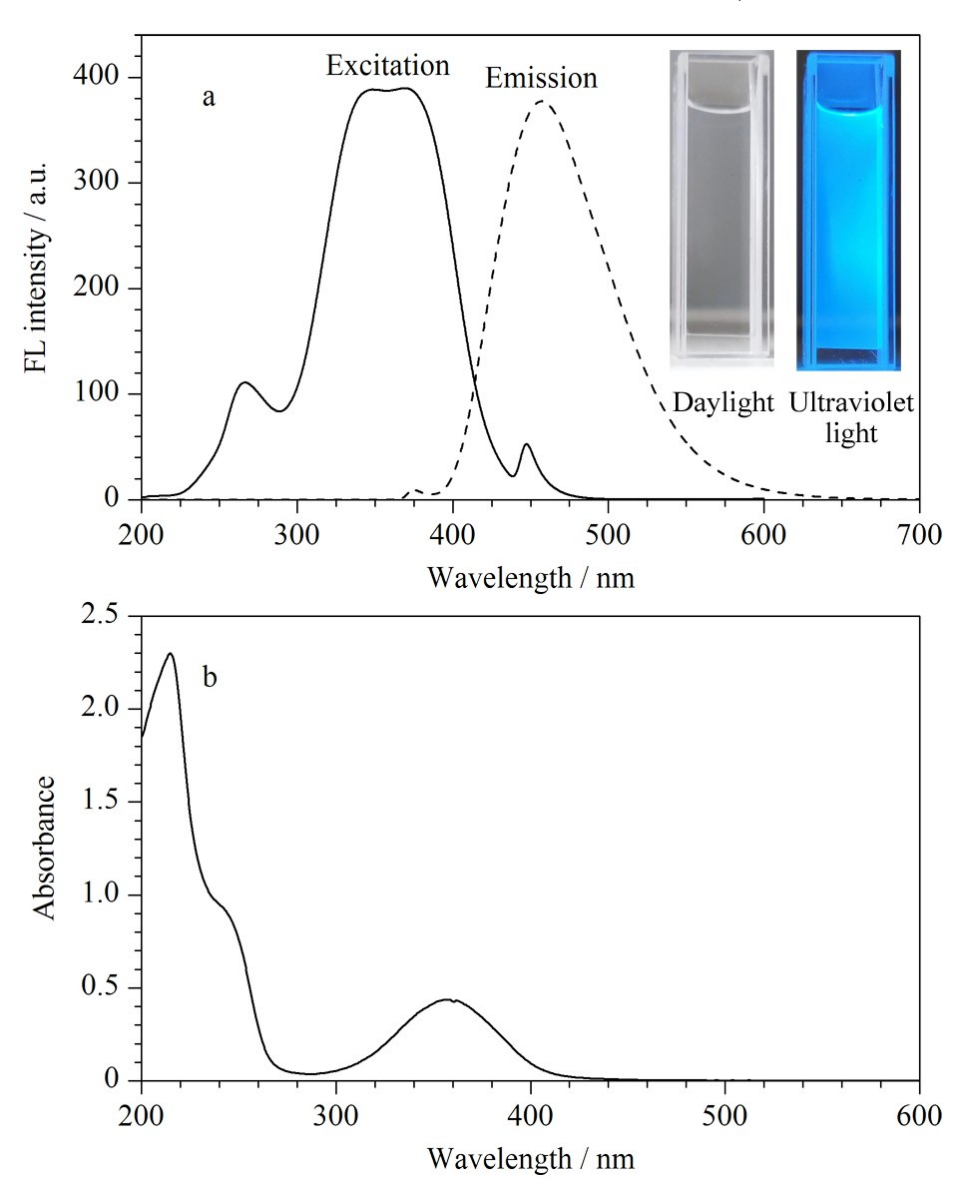
PEI-CDs在超纯水中的(a)荧光光谱图和 (b)紫外-可见光谱图

#### 2.2.2 量子产率的测定

图2为PEI-CDs和硫酸奎宁的PLQY标准曲线。根据式(1)计算得到PEI-CDs的PLQY为45.7%,说明PEI-CDs具有较高的发光效率。

**图 2:**
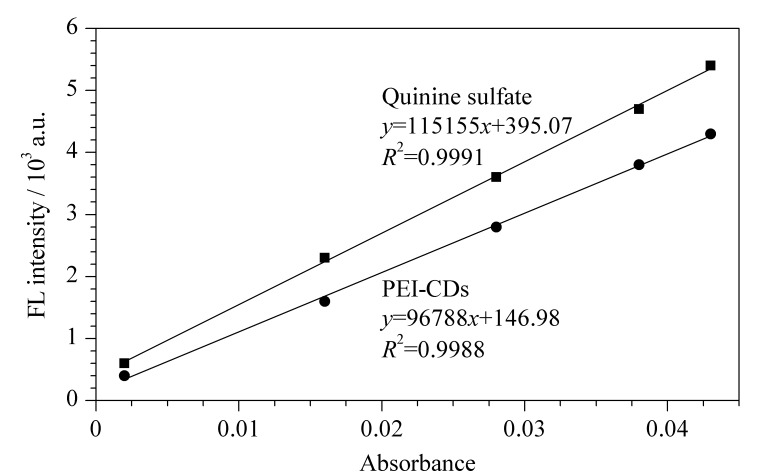
硫酸奎宁和PEI-CDs的量子产率标准曲线

#### 2.2.3 红外光谱表征

图3为PEI-CDs的傅里叶变换红外光谱图。在图3中,3394 cm^-1^处的吸收带对应-NH_2_的伸缩振动,由于在合成PEI-CDs的原料中,只有PEI含有氨基,表明PEI成功修饰到CDs表面。2972 、2852 cm^-1^处的吸收带分别对应-CH_2_的不对称和对称伸缩振动,1700 cm^-1^处的吸收带对应C=O的伸缩振动,1562 、1401 cm^-1^处的吸收带分别对应羧酸盐的不对称伸缩振动和对称伸缩振动,1193 cm^-1^处的吸收带对应C-O的伸缩振动,1072 cm^-1^处的吸收带为C-N的伸缩振动^[23]^。

**图 3:**
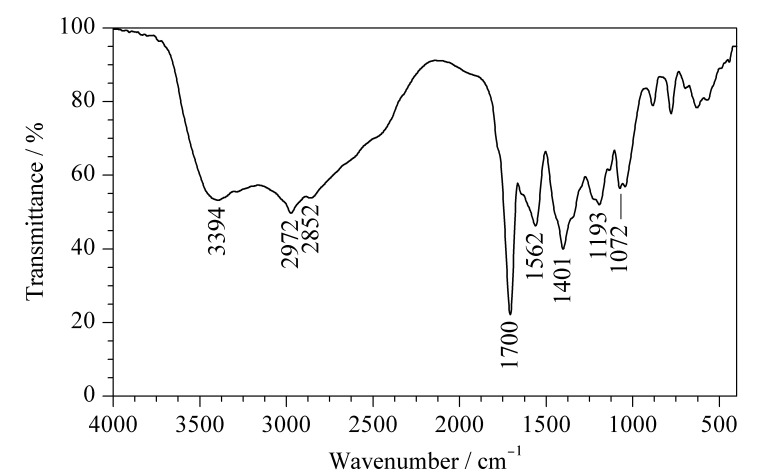
PEI-CDs的傅里变换叶红外光谱图

#### 2.2.4 X射线光电子能谱表征

图4为PEI-CDs的XPS能谱图。PEI-CDs在284、398和531 eV处各有1个峰(图4a),分别对应于C 1*s*、N 1*s*和O 1*s*,表明PEI-CDs由C、N、O 3种元素组成。由表1可见,C、N、O的含量分别为60.15%、18.91%、20.94%。高分辨率的XPS表明,C 1*s*的光谱出现了3个主要峰284.2、285.2和287.3 eV(图4b),分别为C-C/C=C、C-N和C=N/C=O。N 1*s*的光谱(图4c)表明氮元素的杂化方式主要为吡啶型氮(398.7 eV)、氨基氮(399.1 eV)和吡咯型氮(400.9 eV)。O 1*s*的峰值分别为531.2和532.2 eV(图4d),说明O主要以C=O和C-OH/C-O-C的形式存在^[24,25]^。

**图 4:**
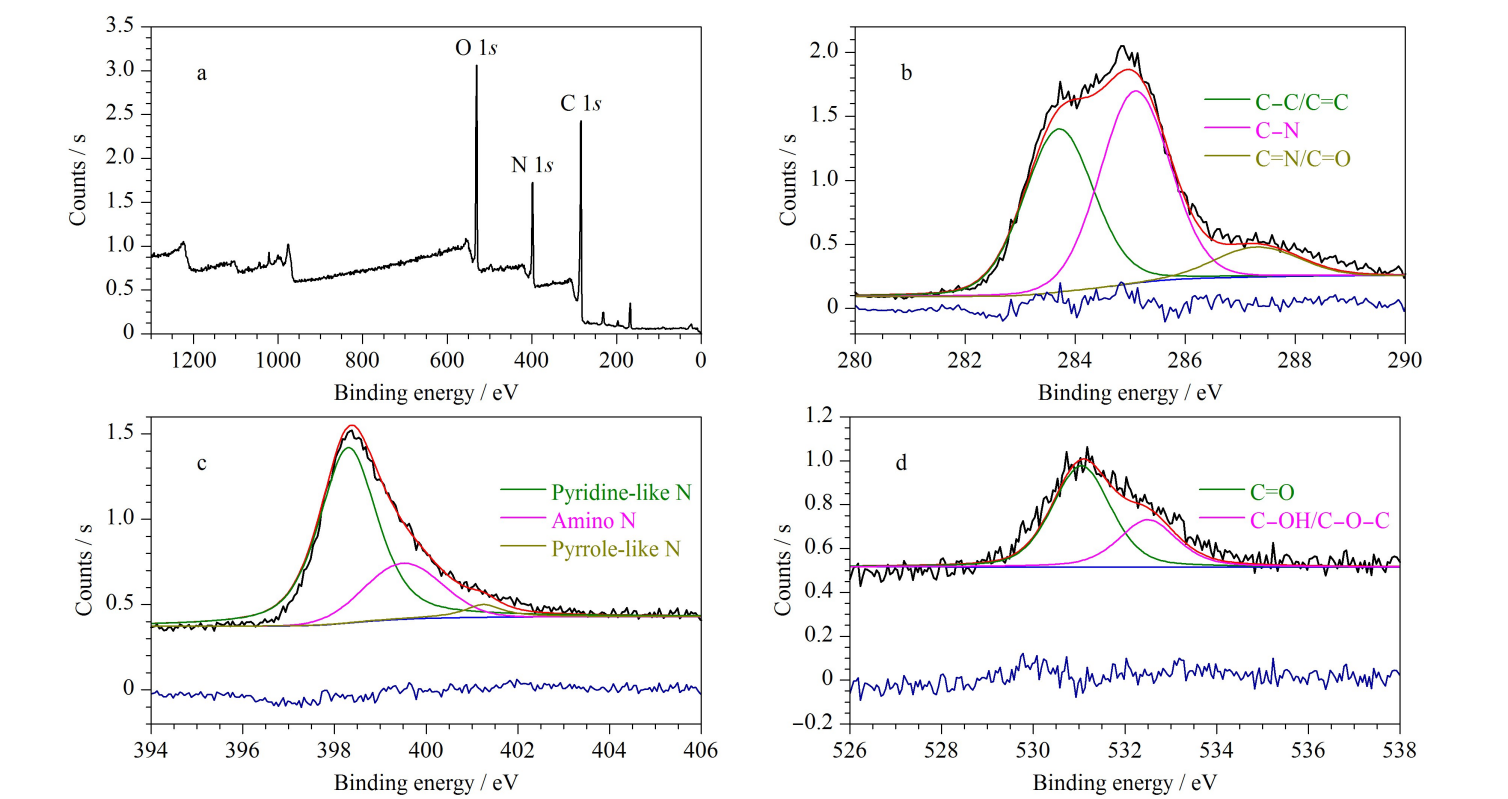
PEI-CDs的XPS能谱图

**表 1:** PEI-CDs的元素分析

Name	Peak/eV	Element content/%
C 1s	284	60.15
N 1s	398	18.91
O 1s	531	20.94

#### 2.2.5 透射电镜表征

TEM是表征纳米材料尺寸和形貌的有效手段。在本实验研究中,利用TEM对PEI-CDs进行表征,如图5所示,实验合成的PEI-CDs为球形,粒径分布较均匀,分散性良好,无明显聚集。粒径分布图显示,PEI-CDs的平均粒径约在4.5 nm左右(图6)。

**图 5:**
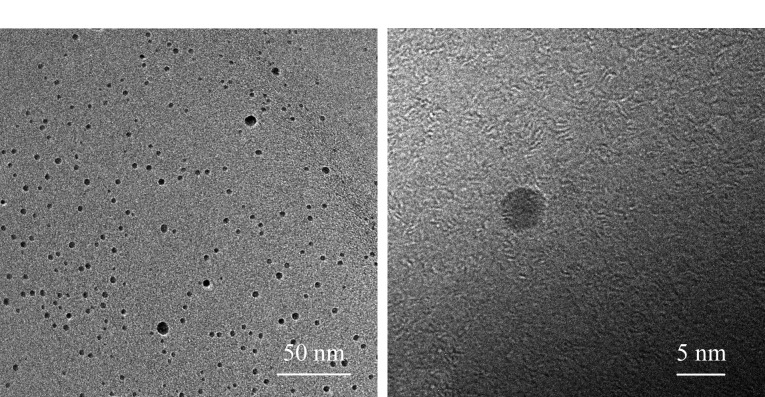
PEI-CDs的透射电镜图

**图 6:**
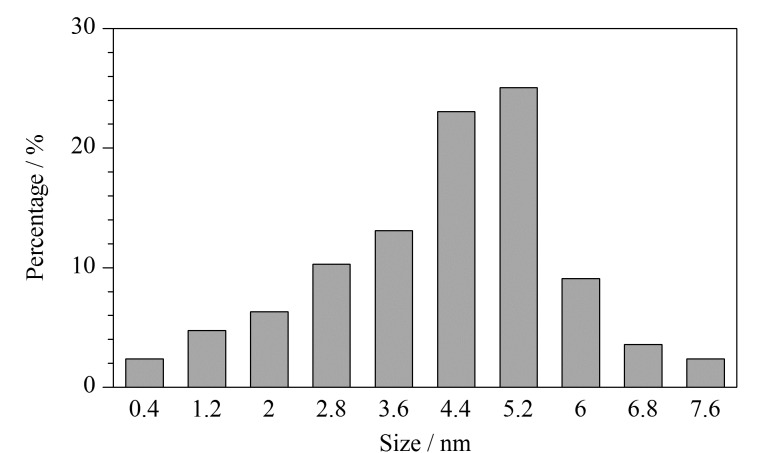
PEI-CDs的粒径分布图

#### 2.2.6 PEI-CDs的稳定性实验

从图7a可以看出,在连续光照18 h期间,PEI-CDs荧光强度基本不变,说明PEI-CDs具有良好的抗光漂白性。从图7b可以看出,PEI-CDs的水溶液放置48 h,其荧光强度基本保持稳定,说明PEI-CDs具有良好的化学稳定性。

**图 7:**
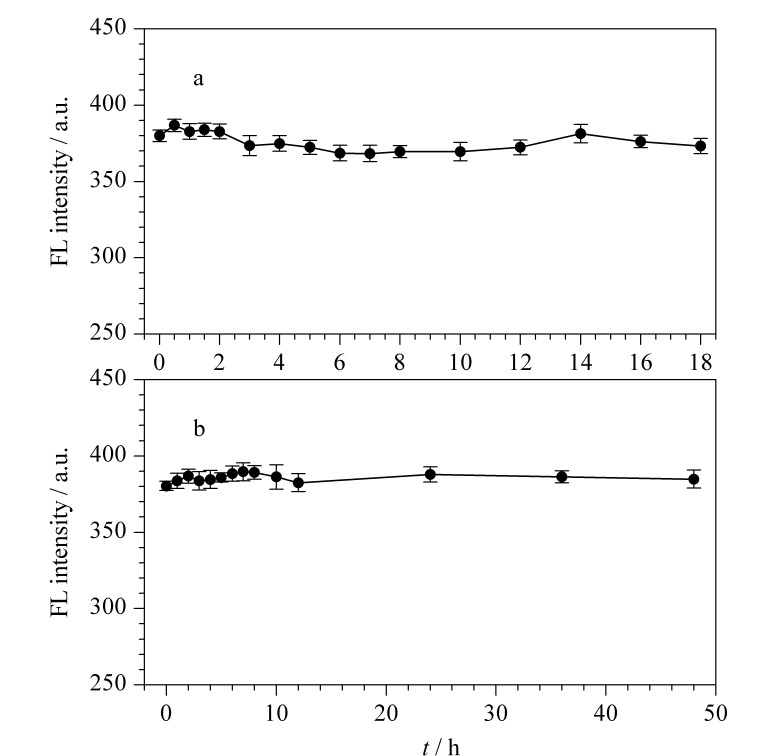
PEI-CDs在(a)紫外光照射下和(b)4 ℃下 荧光强度的变化(*n*=3)

## 3 实验开展

### 3.1 统筹时间合理安排

该开放性实验的开展,分为4个阶段(选题、设计、实施、总结),需要4周时间。其中,实验的选题、设计和总结阶段共2周,主要利用平时的碎片化时间,采用线下线上相结合的形式,进行讨论和指导;实验的实施阶段共2周,利用周末的整块时间,在实验室操作完成。

### 3.2 精心设计自主发挥

在该开放性实验中,材料的合成和纯化是难点,表征是重点。我们结合学生兴趣、科研热点和实验室条件,查阅文献,反复讨论,精心设计,确定方案。

在PEI-CDs的合成中,采用了一步水热法,将制备与掺杂合二为一;在PEI-CDs的纯化中,利用无水乙醇沉淀的方法。整个过程具有简单、快速、经济、绿色的特点,而且制得的PEI-CDs产率高、发光强,能够为后续表征提供充足优质的材料。

在PEI-CDs的表征中,为了防止仪器损坏,必须确保规范操作,所有仪器在使用前要进行培训,使用中要进行指导。当然,作为开放性实验,学生的自主发挥至关重要。我们根据仪器的级别和使用频率“区别对待”,在紫外、荧光和红外光谱的表征中,遵循“学生为主,教师为辅”的原则,学生多次实验、反复练习,达到熟练操作的程度。但是,在TEM和XPS的表征中,由于科研平台的管理要求是提前预约限时使用,因此我们采用“教师详细示范,学生适当操作”的方式,让学生初步了解仪器的使用,重点掌握样品的准备和结果的分析。

### 3.3 严谨认真注重细节

“细节决定成败”,要在规定的时间内完成该开放性实验,必须严谨认真,注意以下事项:(1)将反应原料CA和PEI溶于稀盐酸时,必须超声至完全溶解;(2)高压反应釜必须清洗干净,具体过程为“王水浸泡-清洗-NaOH乙醇洗液浸泡-清洗”;(3)高压反应釜必须拧紧、密封;(4)用无水乙醇洗涤沉淀时,遵循“少量多次,超声分散”的原则,以便获得纯净的PEI-CDs;(5)使用大型仪器时,必须严格遵守操作规范。

## 4 结语

该开放性实验于2023年春季组织实施1次,择优遴选学生4人,在南京医科大学药物分析学系科研实验室开展。通过本实验,激发了学生的学习兴趣,提高了学生的动手能力,培养了学生的创新精神,增强了学生的团队意识。其中两位同学在2023年7月举办的“欧倍尔”杯第七届江苏省大学生化学化工实验竞赛中荣获一等奖、二等奖各1项,另两位同学作为共同主持人获批2023年江苏省高等学校大学生创新创业训练计划项目1项。

但是,由于科研实验室场地的限制,开展规模小,辐射带动作用不充分。后期计划联合南京医科大学药学院的开放实验室,每年培养15位左右学生。该开放实验室于2018年建成,已开展“药物研发链全过程开放实验教学”多年,但其中大型仪器的使用较少,主要是高效液相色谱仪^[26]^。而在“聚乙烯亚胺修饰碳点的合成、纯化与表征”实验中,涉及紫外-可见分光光度计、荧光分光光度计、红外光谱仪、X射线光电子能谱仪和高分辨率透射电镜等精密仪器。两者互为补充,必将助力“懂医精药、善研善成”拔尖创新药学人才的培养。
